# Traditional Korean Medicine Home Care for the Older Adults during the COVID-19 Pandemic in South Korea

**DOI:** 10.3390/ijerph19010493

**Published:** 2022-01-03

**Authors:** Soo-Hyun Sung, You-Sang Baik, Ji-Eun Han, Eun-Jin Lee, Jihye Kim, Minjung Park, Ji-Yeon Lee, Jang-Kyung Park, Jung-Youn Park, Eunkyung Lee

**Affiliations:** 1 Department of Policy Development, National Development Institute of Korean Medicine, Seoul 04554, Korea; koyote10010@nikom.or.kr (S.-H.S.); baikys@nikom.or.kr (Y.-S.B.); jieun2342@nikom.or.kr (J.-E.H.); eunjin6434@nikom.or.kr (E.-J.L.); 2 Department of Korean Medicine Classics, College of Korean Medicine, Kyung Hee University, Seoul 02453, Korea; 3 Research Institute of Korean Medicine Policy, The Association of Korean Medicine, Seoul 07525, Korea; jihyekim1217@gmail.com; 4 National Agency for Development of Innovative Technologies in Korean Medicine, Seoul 07525, Korea; mj.park@nikom.or.kr (M.P.); jyounl@nikom.or.kr (J.-Y.L.); 5 Department of Korean Medicine Obstetrics and Gynecology, Pusan National University, Yangsan 50612, Korea; vivat314@pusan.ac.kr; 6 Department of Health and Welfare, Yuhan University, Bucheon 14780, Korea; park0625@yuhan.ac.kr; 7 Department of Preventive Medicine, Kyung Hee University, Seoul 02453, Korea

**Keywords:** public medical services, community home care, traditional Korean medicine, COVID-19 pandemic

## Abstract

Objectives: The aim of this study was to examine the status of community care services regarding traditional Korean medicine (TKM) for older adults and raise awareness on current opinions and services of TKM institutions. Methods: The National Development Institute of Korean Medicine conducted a survey of 16 local governments by sending official letters through an electronic document system from October 2020 to November 2020. The survey items included basic demographic information and information about TKM service. Results: Eleven (68.8%) of the 16 local governments provided TKM home care services. A total of 136 TKM clinics provided home care services for 598 older adults with musculoskeletal disorders. The number of TKM services provided in five or more local governments were cupping 11 (100.0%), acupuncture 11 (100.0%), education and consulting 10 (90.9%), and moxibustion 9 (81.8%). Moreover, pain (recorded on visual analogue scale) and quality of life significantly improved following TKM services (*p* < 0.001). Conclusions: Covered under medical policy, TKM homecare services could function as a viable alternative for continued medical care disrupted during the coronavirus disease 19 pandemic. In addition, standardisation and legalisation of these services could ensure and improve their efficiency.

## 1. Introduction

Coronavirus disease 19 (COVID-19) was first reported in Wuhan, China in December 2019 and spread rapidly worldwide [1]. In January 2020, the World Health Organization (WHO) declared the COVID-19 outbreak a public health emergency of international concern [2]. On 11 March 2020, the WHO declared this a global pandemic. As of 4 September 2021, 2.8% (218,946,836 persons) of the world’s population was diagnosed with COVID-19, and 2.1% (4,539,723 persons) of confirmed cases had died [3]. The first COVID-19 case in South Korea occurred on 20 January 2020, and as of 4 September 2021, included 0.1% (258,913 persons) of the globally confirmed cases and 0.9% (2315 persons) of confirmed deaths [4].

Every country is focused on managing COVID-19 patients and preventing the spread of disease using fundamental public health principles such as social distancing, hand washing, or mask wearing [5,6,7]. Vaccine development and vaccination are being employed globally, including in South Korea. Some countries, including Singapore and the United Kingdom, with high vaccination rates declared COVID-19 as endemic to concentrate on the management of patients with severe COVID-19 [8,9].

Owing to the focus on a multi-pronged approach involving widespread testing, tracing, and isolation to improve public health during the COVID-19 pandemic, health promotion programs in South Korea were suspended or reduced [10,11]. Due to the increasing number of COVID-19 cases and reduced access to medical facilities, the government has temporarily allowed telemedicine consultations for vulnerable patients (e.g., older adults, patients with mental illness or people with disabilities), and simultaneously promoted community home care [12,13,14]. Community home care is essential for an ageing population and has been introduced as a pilot community project for older adults; since 2019, promotion of Community Care Pilot Projects has been undertaken in 16 local governments involving 242 local governments in cities, districts, and counties in the nation, led by the Ministry of Health and Welfare [14]. The policy on managing health for the older adults requiring public healthcare at home was promoted despite the COVID-19 pandemic.

In cases of home care requiring Western medicine, services regarding examination, testing, education and consulting and referral to the appropriate medical institution is provided for people with impaired mobility [15]. On the other hand, in cases requiring TKM home care, the diagnosis, treatment (e.g., acupuncture, cupping, moxibustion, herbal medicine, or chuna therapy), and education and consulting are performed for older adults with impaired mobility [16]. Before Western medicine was introduced, TKM doctors used acupuncture, electro-acupuncture, pharmacopuncture, herbal medicine, chuna therapy, cupping, moxibustion, and other forms of intervention to treat their patients or prevent diseases [17]. In Korea, 69.0% of the individuals have used TKM, which includes 86.2% of the older adults aged over 60 years [18]. In the case of conventional medicine, home care is provided to monitor the health status of a patient and refer them to a medical institution, whereas, in the case of TKM, home care involves consulting and education as well as treatment. Thus, in TKM, it is possible to provide actual treatment during home visits due to the TKM characteristics of performing treatment with simple tools or hands.

Most of the published studies concerning home care during the COVID-19 pandemic have focused on the conventional medical approach [19,20,21]. This is the first study to investigate the importance of TKM home care for older adults amid an increase of COVID-19 cases. The results of this study will contribute to our understanding of gaining access to vulnerable populations during pandemics and raise awareness on current guidelines and services offered by TKM institutions [22]. In addition, these findings could contribute to the government’s policy of introducing TKM services in the community and thus help determine their utilization.

## 2. Materials and Methods

### 2.1. Scope of the Survey

The study was conducted in 16 localities (cities or districts) offering pilot programs for community care in South Korea.

### 2.2. Development of the Questionnaire

The questionnaire was developed by three researchers (including one TKM specialist with 10+ years of clinical experience, one TKM PhD holder with 10+ years of experience in the field, and one TKM policy researcher who is a project investigator in the TKM Community Care Monitoring and Evaluation Project) and was based on findings from previous studies [23,24,25]. The draft questionnaire was reviewed by three researchers (M.P., J.K.P. and E.L.). Based on the reviewers’ comments, the research team had further discussions to finalise the questionnaire.

The questionnaire consisted of three sections assessing the TKM service offered incorporating questions on background information, effectiveness of the service, and patient satisfaction. The background information consisted of 11 items including area, type of care, participating institution, participant (group, diseases treated, number of patients), intervention (type of intervention used, treatment duration, number of sessions, and cost), and number of assistants required. The six items (participating institution, number of participants, treatment duration, number of sessions, cost, and number of assistants) were organised with the declaration enabled by indicating the unit in the empty section (see Additional File S1 in Supplementary Material for the final version of the questionnaire).

This study used a dynamic age structure model as the basis for classifying the participant’s area. This is a method for estimating the age structure of the participant’s area through its conversion into functions of the rates of birth, mortality, and increase in age [26]. The area types are based on the dynamic age structure model and are categorised into six groups. First is a metropolis with an active population reproduction and influx, second is a metropolis with a large population despite poor reproduction and influx, third is an urban-rural complex experiencing large-scale population influx and urbanisation due to the development of the rural area, fourth is an area with characteristics of the urban-rural complex but showing characteristics of an aging population, fifth is an area experiencing a new, small influx of population but showing features of the old rural area, and sixth is an area with little new influx of population and showing features of the old rural area [26].

To assess the effectiveness of the TKM service, the questionnaire had two items and was formatted to enable the easy entry of information. A visual analogue 10-point scale (VAS) was used to score pain and the 5-level quality of life (EQ-5D) tool was used to assess quality of life before and after receiving the TKM treatment. Five items were used to assess for satisfaction with the TKM service and were organised to enable easy entry for each participant. The surveys for assessing the effectiveness of and satisfaction with the TKM services, were distributed by the Association of Korean Medicine to the 16 areas practicing TKM in advance in early 2020.

### 2.3. Survey Method

The National Development Institute of Korean Medicine (NIKOM) conducted a survey of the 16 localities by sending official letters through an electronic document system from October 2020 to November 2020. In addition, NIKOM researchers contacted the person in charge of each community care pilot program. They then explained the aims of the research, the questionnaire development procedure, and the survey methods.

Local governments selected individuals with poor economic conditions, along with those with mental health issues and the older adults living alone as targets for integrated community care services. Among the selected subjects, TKM homecare services were provided to the older adults with reduced mobility who requested TKM. Therefore, the developed questionnaire was requested by the person in charge of the local government based on the information of the TKM homecare service target and were completed by the officers who were responsible for the Community Care Pilot Projects to help depict the current situation as accurately as possible. The researchers did not request replies if there were no TKM-related programs offered. The researchers reviewed the completed questionnaires received by NIKOM and requested any missing data from the localities.

For the effectiveness (VAS and EQ-5D) and satisfaction of the TKM service provision components, anonymised spreadsheet data were received from the Community Care Pilot Project staff. Data were requested to be submitted to a local government official if there were pre- and post-evaluation results for patients treated by TKM doctors who administered treatment during home visits.

### 2.4. Study Selection

**Figure 1:**
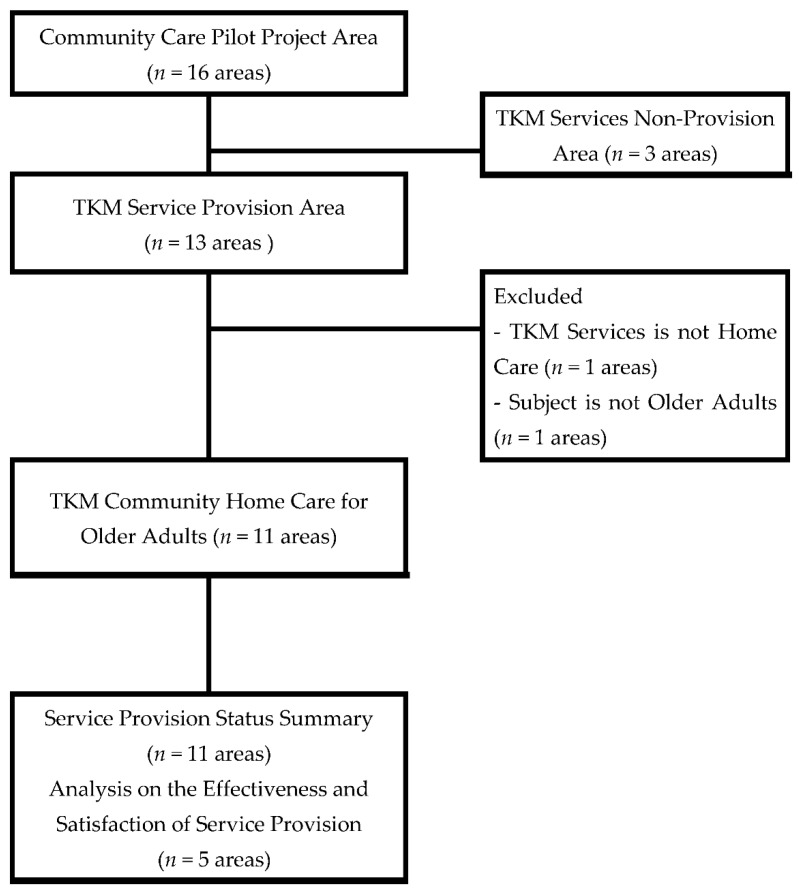
Flow chart of inclusion and exclusion of respondents from survey data.

The survey data were secured from the 13 TKM service-providing areas of the 16 local governments. Among these, one area providing home care TKM services for people with disabilities and one area providing a non-home care service for the older adults were excluded. The study was conducted on survey data from the 11 areas providing TKM home care services for the older adults. The selection process for participants is shown in Figure 1.

### 2.5. Statistical Analysis

Data were divided by area characteristics (area, area type, total population, older adult population rate, single older adult household rate, unmet medical need rate) and service characteristics type of care, number of participating institutions, diseases treated, number of participants, intervention types, treatment duration, number of sessions, cost, and number of assistants). To evaluate the effectiveness of TKM services, 10 mm VAS and EQ-5D data were collected from the questionnaire. Further, data on satisfaction with the TKM service were also collected. Korea Plus Statistics (Embedded on SPSS Statistics 26, IBM Corp., Armonk, NY, USA) for Windows was employed for the statistical analysis. Data are expressed as mean ± standard deviation (SD). The effect of the treatment in each group was evaluated using paired t-tests applied to the pre- and post-treatment scores.

## 3. Results

### 3.1. Basic Characteristics of Areas

Table 1 presents the characteristics of the local governments which participated in this survey. Four (36.4%) were for type 1 area (metropolis with active population reproduction and influx), five (45.4%) for type 2 area (metropolis with poor population reproduction and influx), and two (18.2%) for type 4 area (urban rural complex type with an aging population).

In view of the dynamic age structure and other demographic and health characteristics of the participants’ areas, we found that the single older adult household rate and the unmet medical need rate was high in type 1 areas (metropolis type with active population reproduction and influx). In particular, the unmet medical need rates in Cheonan Chungnam and Ansan Gyeonggi were 17.6% and 9.6%, respectively—which were higher than those of other areas—and the number of doctors per medical institution was 3.3 and 2.2, respectively, showing a level similar to the median (3 persons) for all participant areas. Accordingly, type 1 areas had a health and medical service provision infrastructure that was relatively similar to that of other areas of high medical demand; however, the delivery system is insufficient. 

The older adult population rate, the single older adult household rate, and the unmet medical need rate were high in type 2 areas, and the number of medical doctors in each institution was lower than the median (3 persons) for all participant areas except for Busanjin Busan (5.2 persons) and Seo-gu Gwangju (3.6 persons). Accordingly, the type 2 areas also had a health and medical service provision infrastructure that is relatively similar to that of other areas of high medical demand such as type 1 areas but the delivery system was insufficient.

Further, the older adult population rate and the single older adult household rate was very high in type 4 areas (urban rural complex type with aging population) compared to other areas; however, the unmet medical need rate was at a level close to the median (7.0%) of all participant areas, with the number of medical doctors per institution not reaching the median (3 persons) of all areas. Accordingly, it can be interpreted that the demand was low due to insufficient health and medical service provision infrastructure, despite the necessity for medical services.

### 3.2. Current Provision of TKM Community Home Care Service

**Figure 2:**
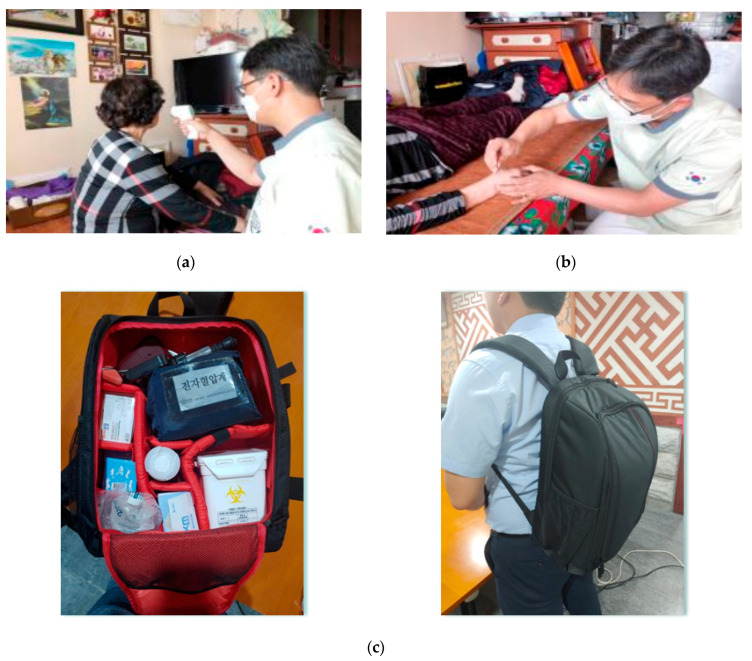
The process of TKM community home care. TKM: Traditional Korean medicine. (**a**) Body temperature check before treatment, (**b**) Acupuncture intervention, (**c**) TKM home care bag. The home care bag contains a manometer, acupuncture needles, moxibustion, cupping devices, herbal medicines, a waste collection box, thermometer, and hand sanitiser.

**Figure 3:**
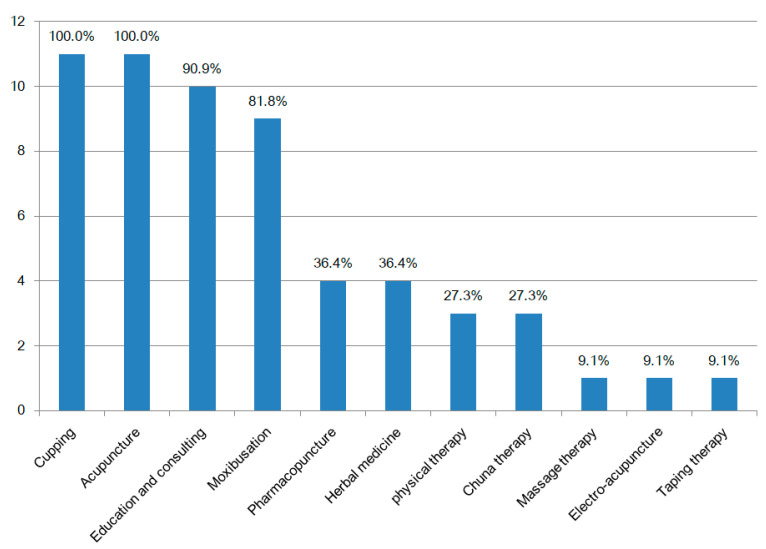
Current status of community home care of TKM Intervention. TKM: Traditional Korean medicine.

The provision of TKM community home care services is shown in Table 2. The processes involved and the home care bag are shown in Figure 2. A total of 136 TKM clinics provided home care services for 598 older adults with musculoskeletal disorders. The number of TKM services provided in five or more areas were cupping, 11 (100.0%); acupuncture, 11 (100.0%); education and consulting, 10 (90.9%); and moxibustion, 9 (81.8%) (Figure 3). The treatment period ranged from 2 to 12 months, with 4 to 12 sessions per patient. The session/total treatment cost ranged from 83.3 to 166.7 United States Dollar (USD). All service costs were paid by the local governments without setting any user charges for older adults. In the case of home care services, an assistant accompanied a TKM doctor in seven of the local government areas, and a TKM doctor alone visited the patient in four local government areas. 

### 3.3. Effectiveness of and Satisfaction with TKM Service Provision

We found that pain (VAS) and quality of life (EQ-5D) improved significantly after TKM (*p* < 0.001, Table 3). The satisfaction with the TKM service was found to be 4.5 points or more for all five questions. The question enquiring whether the TKM service was helpful to health scored the highest. The convenience of the process scored lowest (Table 4).

## 4. Discussion

This study is a government-led survey evaluating the provision and effectiveness of the TKM service available to older adults as a public service during the COVID-19 pandemic as well as the satisfaction levels experienced by the recipients of this service. In particular, it is the summation of the combined results of a survey conducted by local governments operating Community Care Pilot Projects implemented by the Korean government. Hence, this study provides evidence for effective implementation of TKM community care provision policy [27,28]. Remarkably, several TKM institutions (*n* = 136) participated in this project, providing a TKM community home care program for a total of 598 people. As such, the TKM service provided in a home setting even during the COVID-19 pandemic imparts insights on the type of public health service that should be provided in the future during pandemics or similar infectious disease situations.

The strategy of social distancing, introduced to manage the COVID-19 pandemic, radically changed the daily lives of older adults [29]. All health improvement projects at public health centre were suspended, and patients avoided over-crowded hospitals due to the fear of infection [30]. COVID-19 pandemic caused 68.8% of older adults in the United States of America to cancel doctor’s appointments, and almost half of them to cancel operations or treatment [31]. In South Korea, the number of health insurance claims by medical institutions decreased by 13.7% in 2020 compared with 2019; the number decreased by 4.3% for the older adults. [32]. Hence, the medical accessibility of this group with complex medical demands was lowered considerably. Additionally, the quality of life of older adults with chronic diseases during the pandemic was significantly lower than that of those without chronic disease [33]. Healthcare for older adults with chronic disease was an unmet need during the pandemic.

Through the TKM Community Care Pilot Projects, necessary healthcare could be conveniently provided via home visit by medical personnel to vulnerable older adults with high unmet needs without potentially exposing them to COVID-19 infection.

Regions corresponding to type 1 showed a lower number of doctors per 1000 individuals compared to other regions. In Cheonan Gyonggi, the proportions of older adults living alone and unmet medical care were found to be high, even though the average number of doctors per 1000 individuals was above the average in the entire region. Most of the regions corresponding to Type 2 had low unmet medical care rates; however, in Buk-gu Busan, the older adult population, the older adults living alone, and unmet medical care rates were all high. We tried to identify the difference in TKM home care services according to the regional types or characteristics, but it was difficult to identify the distinct characteristics of each of the three types. In addition, for regions with different characteristics within the same region type, it is necessary to establish the TKM home care services model based on in-depth analysis and demand survey. Nevertheless, this data can be used to determine the region whose model can be used as a reference in the process of implementation of TKM homecare services nationwide in the future.

There are some limitations to this study. First, it is necessary to improve the questionnaire method and type of questions in future studies. As this survey included responses obtained only from project managers of each local government according to the limitations of the Personal Information Act, detailed and accurate information (e.g., gender, age, academic background, or health status) of the TKM homecare services recipients was not available. In the future, in the process of selecting targets for TKM homecare services, it is necessary to improve the business methodology such that personal information can be effectively obtained for specific purposes such as policy development or research. Second, statistically significant improvements in pain and quality of life were seen after the TKM service. However, these results were derived from incomplete data (VAS for 126 persons and EQ-5D for 34 persons) of the total 598 subjects. This study can be considered a pilot study, evaluating the effect of TKM home care services. It was extremely challenging during the pandemic to plan large-scale evaluation studies. Even though we were able to perform pre- and post- treatment comparisons in our study, lack of a control group and having a small number of respondents limited the quality of our results. Third, there were structural differences in the TKM services provided by the 11 regions (e.g., service type, duration, or skill level of TKM doctors). A certain level of standardization is required to guarantee the specific quality of TKM services provided in each region and ensure service costs at the national insurance level. To this end, at the central government level, a system that can monitor, evaluate, and adjust regional project progress is required.

To provide the TKM homecare service within the community care system, it is necessary to develop a standard manual for TKM home care for older adults. Acupuncture and cupping were used in all areas but the remaining TKM interventions varied among areas. The standard manual should include educational materials, the consulting method employed, materials needed, and so on.

## 5. Conclusions

The continued spread of COVID-19 has prompted each country to undertake strong remedial measures such as social distancing or lockdowns. It is expected that this will lower access to medical care, resulting in an increase in the proportion of unmet medical care and deterioration of the health of the older adults. In this respect, TKM home care could prove to be a good alternative to conventional medical approach in the regions where coronavirus infections persist.

In the future, it is necessary to standardise care (e.g., the diseases treated, treatment method, period, and frequency, and clarify assistant standards and roles) and legislate (e.g., the treatment procedure during a pandemic and the response to the occurrence of a medical accident) to ensure a high-quality service.

## Figures and Tables

**Table 1:** Demographics of participant’s areas.

Areas	Area Type	Area Characteristics				
		Total Population (Persons)	Older Adult Population Rate	Single Older Adult Household Rate	Unmet Medical Need Rate	Number of Medical Institution Medical Doctors * (Persons)
Median	-	450,168	14.0%	20.0%	7.0%	3.0
Seo-gu Gwangju	2 type	300,975	13.4%	20.3%	7.1%	3.6
Buk-gu Busan	2 type	291,132	15.8%	23.2%	14.1%	2.4
Busanjin-gu Busan	2 type	357,880	18.8%	21.6%	4.4%	5.2
Ansan Gyeonggi	1 type	650,918	10.2%	19.9%	9.6%	2.7
Bucheon Gyeonggi	2 type	829,996	12.5%	18.3%	7.8%	3.0
Namyangju Gyeonggi	1 type	701,830	13.3%	14.6%	3.4%	1.5
Cheonan Chungnam	1 type	652,268	10.3%	19.4%	17.6%	3.3
Jincheon Chungbuk	4 type	81,084	17.1%	21.7%	6.8%	1.7
Gimhae Gyeongnam	2 type	542,455	10.6%	20.3%	6.5%	1.9
Suncheon Jeonnam	4 type	279,598	15.1%	21.6%	9.5%	2.7
Seoguipo Jeju	1 type	181,584	18.6%	17.0%	5.6%	1.7

* Number of medical doctors per 1000 persons.

**Table 2:** Current Provision of TKM Home Care Services.

Areas	Type of Care (Number of Participating Institutions)	Treatment Diseases/Number of Subject	Intervention			Number of Assistants
			Type of Used Intervention	Treatment Duration, Sessions	Cost	
Seo-gu Gwangju	Home care (*n* = 7)	Old adults (Musculoskeletal disorder ^(^^1)^/*n* = 60)	Acupuncture Cupping Taping Exercise therapy Chuna manual therapy ^(^^3)^ Education and consulting	9 month, 12 sessions per person	100 USD (User charge 0 USD)	*n* = 0
Cheonan Gyeonggi	Home care (*n* = 25)	Old adults (Musculoskeletal disorder ^(^^1)^/*n* = 73)	Acupuncture Moxibustion ^(^^2)^ Cupping Chuna manual therapy ^(^^3)^ Herbal medicine Massage	12 month, 12 sessions per person	95.8 USD (User charge 0 USD)	*n* = 25 (Nurse’s aide)
Seoguipo Jeju	Home care (*n* = 6)	Old adults (Musculoskeletal disorder ^(1)^/*n* = 42)	Acupuncture Moxibustion ^(2)^ Cupping Education and consulting Herbal medicine	12 month, 8 sessions per person	95.8 USD (User charge 0 USD)	*n* = 0
Ansan Gyeonggi	Home care (*n* = 32)	Old adults (Musculoskeletal disorder ^(^^1)^/*n* = 105)	Acupuncture Moxibustion ^(^^2)^ Cupping Pharmacopuncture Exercise therapy Education and consulting	6 month, 8 sessions per person	83.3 USD (User charge 0 USD)	*n* = 50 (Nurse’s aide or community service centre staff)
Jincheon Chungbuk	Home care (*n* = 3)	Old adults (Musculoskeletal disorder ^(^^1)^/*n* = 58)	Acupuncture Moxibustion ^(^^2)^ Cupping Chuna manual therapy ^(^^3)^ Education and consulting	6 month, 8 sessions per person	100 USD (User charge 0 USD)	*n* = 3 (Nurse’s aide)
Busanjin-gu Busan	Home care (*n* = 13)	Old adults (Musculoskeletal disorder ^(^^1)^/*n* = 35)	Acupuncture Moxibustion ^(^^2)^ Cupping Education and consulting	8 month, 8 sessions per person	166.7 USD (User charge 0 USD)	*n* = 2 (Nurse’s aide) *n* = 12 (Community service centre staff)
Buk-gu Busan	Home care (*n* = 16)	Old adults (Musculoskeletal disorder ^(^^1)^/*n* = 9)	Acupuncture Moxibustion ^(^^2)^ Cupping Pharmacopuncture Education and consulting	4 month, 4 sessions per person	166.7 USD (User charge 0 USD)	*n* = 3 (Nurse’s aide) *n* = 13 (Community service centre staff)
Bucheon Gyeonggi	Home care (*n* = 19)	Old adults (Musculoskeletal disorder ^(^^1)^/*n* = 80)	Acupuncture Moxibustion ^(^^2)^ Cupping Herbal medicine Chuna manual therapy ^(^^3)^ Education and consulting	2 month, 12 sessions per person	100 USD (User charge 0 USD)	*n* = 10 (Community service centre staff)
Gimhae Gyeongnam	Home care (*n* = 2)	Old adults (Musculoskeletal disorder ^(^^1)^/*n* = 21)	Acupuncture Electroacupuncture Pharmacopuncture Moxibustion ^(^^2)^ Cupping Herbal medicine Education and consulting	12 month, 8 sessions per person	100 USD (User charge 0 USD)	*n* = 0
Suncheon Jeonnam	Home care (*n* = 4)	Old adults (Musculoskeletal disorder ^(^^1)^/*n* = 50)	Acupuncture Pharmacopuncture Cupping Education and consulting	12 month, 4 sessions per person	98.3 USD (User charge 0 USD)	*n* = 1(Social worker) *n* = 1 (Community service centre staff)
Namyangju Gyeonggi	Home care (*n* = 9)	Old adults (Musculoskeletal disorder ^(^^1)^/*n* = 65)	Acupuncture Moxibustion ^(^^2)^ Cupping Exercise therapy Education and consulting	2 month, 6 sessions per person	125 USD (User charge 0 USD)	*n* = 0

USD: United States Dollar/1 USD = 1200 South Korean Won, TKM: traditional Korean medicine. ^(1)^ Musculoskeletal disorder: disc related disease (herniation of intervertebral disc, spinal stenosis), Osteoarthritis, pain and sprain in neck, shoulder, back, knee, or ankle. ^(2)^ Moxibustion: treatment that stimulates acupuncture points with heat generated from burning herbal medicines containing mugwort (Artemisia vulgaris). ^(3)^ Chuna therapy: manual therapy used to balance orthopaedic structure and function by applying thrust, mobilization, distraction of the spine and joints, visceral manipulation, soft tissue release, craniosacral therapy, and the diaplasis technique along the meridian.

**Table 3:** Effectiveness of TKM Services.

Outcome	N (Number of Persons)	Pre (Mean ± SD)	After (Mean ± SD)	*p*-Value
VAS	126	7.74 ± 1.90	6.13 ± 2.30	0.000 **
EQ-5D total score	34	11.09 ± 2.17	10.07 ± 2.19	0.000 **
Q1 score	34	2.29 ± 0.46	2.15 ± 0.44	0.000 **
Q2 score	34	1.91 ± 0.79	1.81 ± 0.80	0.000 **
Q3 score	34	2.21 ± 0.64	2.09 ± 0.62	0.000 **
Q4 score	34	2.50 ± 0.51	2.21 ± 0.54	0.003 *
Q5 score	34	2.18 ± 0.76	1.85 ± 0.61	0.000 **

* EQ-5D: Euro-Quality of Life-5 Dimension; VAS: visual analogue scale; Q1: Question about Mobility, Q2: Question about Self-care, Q3: Question about Usual activites, Q4: Question about Pain/discomfort, Q5: Question about Anxiety/depression; * *p* < 0.01, ** *p* < 0.001.

**Table 4:** Satisfaction with TKM Service Provision.

Questionnaire	N (Number of Persons)	Minimum	Maximun	Average (Mean ± SD)
Q1. Was the TKM service provide sufficient information?	60	1	5	4.37 ± 0.82
Q2. Was it convenient to apply for and participate in the TKM service?	60	1	5	4.30 ± 0.93
Q3. Was the TKM service helpful to health?	60	3	5	4.76 ± 0.47
Q4. Did your ability to manage your condition yourself improve after the TKM service?	60	3	5	4.51 ± 0.57
Q5. Are you willing to recommend the TKM service to others?	60	3	5	4.65 ± 0.58

N: number of subject, TKM: Traditional Korean medicine, Q: Question.

## Data Availability

The data will be made available upon reasonable request.

## Supplementary Materials

The following are available online at https://www.mdpi.com/article/10.3390/ijerph19010493/s1, Additional File S1: Current Status of Community Care Services of Traditional Korean Medicine for Older Adults: A Survey.
