# Supplementary material for: Traditional Korean Medicine Home Care for the Older Adults during the COVID-19 Pandemic in South Korea
Source: Int J Environ Res Public Health. 2022 Jan 3;19(1):493. doi: 10.3390/ijerph19010493 (PMC8744707; doi:10.3390/ijerph19010493)
Supplement: Supplementary file 1 [file ijerph-19-00493-s001.zip › ijerph-1464633-supplementary.pdf]

Current Status of Community Care Services of Traditional Korean Medicine for Older Adults: A Survey

NIKOM

[Objectives]

By collecting data on the current community provision of traditional Korean medicine (TKM) for the older adults, we intend to provide evidence for the development of TKM community services as an integrated part of a community care system.

[Targets]

The 16 local governments promoting the community care pilot projects

[Items]

The survey items comprised two parts:

1. Background information: Area, area type, area characteristics (total population, older adult population rate, single older adult household rate, unmet medical need rate, and number of doctors per institution)

2. TKM service: TKM service type, institution providing service, participants and their number, type of intervention offered, provision period, provision frequency, provision cost, and use of assistants

[Instructions]

The National Development Institute of Korean Medicine (NIKOM) intends to conduct a survey to investigate the status of TKM community care services for the older adults. Your answers will contribute to the establishment of TKM services as an integrated part of a community care system. The contents of this questionnaire will be used in a statistical analysis for academic purposes only, and any personal information will remain strictly confidential other than for this analysis.

[PART 1. Basic Characteristics of areas]

Areas	Area type[1)]	Area characteristics				
		Total population	Elderly population rate[2)]	Single elderly household rate[3)]	Unmet medical need rate	Number of medical institution medical doctors[4)]

1) Select the area type from the following 6 types.

1. Type 1: Metropolis with active population reproduction, and influx
2. Type 2: Metropolis with poor population reproduction and influx
3. Type 3: Urban rural complex with large-scale population influx
4. Type 4: Urban rural complex with an aging population
5. Type 5: Old rural area with population influx
6. Type 6: Old rural area

2) Rate of those aged 65 years or older in the total population

3) Rate of the single older adult household of those aged 65 years or older

4) Number of medical doctors per 1,000 persons

[PART 2. Current Status of TKM services]

1. What is the TKM service type?

(1) Home Care (2) Public Use Facility (e.g. the senior citizen centre or the welfare centre) (3) TKM Clinics

(4) Other: _______________

2. How many institutions offer TKM services? (______________units)

3. Who are the participants that receive the TKM service, and how many participants are there?

(1) Older Adults (n =) (2) People with Disabilities (n =) (3) People with mental illness (n =)

(4) Other: _______________ (n =)

4. What is the intervention type used for the TKM service?

(1) Acupuncture (2) Moxibusion (3) Cupping (4) Herbal Medicine (5) Chuna Therapy (6) Pharmacopuncture

(7) Exercise Therapy (8) Education and Consulting (9) Other: _______________

5. How long has TKM been offered for? 00 (month) 0000 (year) to 00 (month) 0000 (year)

(Total_______________months)

6. What is the TKM service frequency per person? (_______________sessions per person)

7. What is the TKM service cost per person?

(1) (Total___________South Korean Won per person) (2) (User Charge___________South Korean Won per person)

8. Are assistants providing the TKM service?

(1) Yes (n =) (2) No
